# Related variations: A novel approach for detecting patterns of regional variations in healthcare utilisation rates

**DOI:** 10.1371/journal.pone.0287306

**Published:** 2023-06-22

**Authors:** Jan Håkon Rudolfsen, Jan Abel Olsen

**Affiliations:** 1 Department of Community Medicine, University of Tromsø, Tromsø, Norway; 2 Centre for Health Economics, Monash University, Melbourne, Victoria, Australia; 3 Division of Health Services, Norwegian Institute of Public Health, Oslo, Norway; Houston Methodist Academic Institute, UNITED STATES

## Abstract

Regional variations in healthcare utilisation rates are ubiquitous and persistent. In settings where an aggregate national health service budget is allocated primarily on a per capita basis, little regional variation in *total* healthcare utilisation rates will be observed. However, for *specific* treatments, large variations in utilisation rates are observed, iymplying a substitution effect at some point in service delivery. The current paper investigates the extent to which this substitution effect occurs *within or between* specialties, particularly distinguishing between emergency versus elective care. We used data from Statistics Norway and the Norwegian Patient Registry on eight somatic surgeries for all patients treated from 2010 to 2015. We calculated Diagnosis-Related Group (DRG) -weight per capita in 19 hospital regions. We applied principal component analysis (PCA) to demonstrate patterns in DRG-weight, annual relative changes in DRG-weight, and DRG-weight production for elective care. We show that treatments with similar characteristics cluster within regions. Treatment frequency explains 29% of the total variation in treatment rates. In a dynamic model, treatments with a high degree of emergency care are negatively correlated with treatments with a high degree of elective care. Furthermore, when considering only elective care treatments, the substitution effect occurs *between* specialties and explains 49% of the variation. When designing policies aimed at reducing regional variations in healthcare utilisation, a distinction between elective and emergency care as well as substitution effects need to be considered.

## 1. Introduction

Regional variation in healthcare utilisation rates is a well-known phenomenon. An immense body of literature has documented those regional variations to be ubiquitous and persistent [1,2]. While mean utilisation rates differs across healthcare systems, the extent of regional variation in utilisation are remarkably similar [3]. Moreover, relatively high, or low regional utilisation rates of healthcare services are persistent over time. This phenomenon is referred to as ‘surgical signatures’, which explain 55–93% of regional variation in utilisation rates [4]. The persistency of high or low rates for a given treatment has been ascribed to physicians who specialise in sets of treatments and have a bias towards providing them [5]. This bias is often referred to a ‘preference sensitive variation’, and is along with supply constrictions, or ‘supply sensitive variation’, thought to be major drivers of variation in utilisation of healthcare services [6]. When the regional variation is not due to population need, it is unwarranted and should be eliminated.

In the context of a healthcare system with strictly regulated and fixed budgets in the short run, an oversupply of some practitioner-preferred treatments would imply corresponding undersupply elsewhere–e.g., relatively high provision of outpatient care results in reduced provision of inpatient clinical care, or primary care. Otherwise, the budget would exceed the allocated amount. However, only a few studies have investigated substitution effects across specific treatments in a hospital setting. Phelps and Mooney (1993) found negative correlations between Intensive Care Unit admissions and elective admissions, but positive correlations for surgical versus medical treatment of specific conditions [7]. Reschovsky et al. (2014) considered correlations in mean cost per episode for ten clinical conditions. The highest correlation coefficients were found between COPD/asthma versus bacterial lung infection (correlation = 0.63)–the latter being a common complication of the former. The second highest correlation, however, was neck/back surgery versus knee/lower leg surgery (correlation = 0.4), both performed by the same specialist [8]. The theoretical explanation being that as prices are fixed to the national average cost, cost-sensitive providers will have an incentive to provide excess services in which they have a comparative advantage–i.e., if a hospital can provide a treatment at a lower cost than the national average cost, they will be inclined to do just that, as it will allow for the hospital to increase their budgets. If this is the case, then fewer resources will be available for other treatments.

To the best of our knowledge, no previous studies have considered the role of elective versus emergency care in this context. It is well established that elective care is the area where we expect to find regional variation in treatment rates [9]. Emergency care patients often exhibit more distinguished symptoms [10], leaving less room for physician bias. Moreover, the provision of elective care treatments is influenced by patient preferences [11–13] and hospital financial incentives [14–16].

This paper provide new knowledge on patterns in regional variations. As budgets are fixed, provision of a given treatment reduces resources available to provide all other treatments. As hospitals are cost-sensitive, and respond to financial incentives, a substitution effect must be present. The aim of this paper is to investigate whether the substitution effect in treatment rates occurs *within* or *across* medical specialties, and the extent to which elective care differs from emergency care.

## 2. Study setting and included treatments

Norwegian specialist care is fully financed by the state in a national health service, offering a unique institutional context to consider treatment patterns. Municipalities are responsible for primary care. In order to access specialist care, patients need a referral from their general practitioner (GP).

Once referred to specialist care, patients are free to choose the treating hospital (public or private specialist contracted by the state). According to national guidelines, surgeons should prioritise patients on the waiting list based on an assessment of their expected health gains and the severity of their condition.

Specialised care is organised in four regional hospital trusts that are financed by a combination of block grants and prospective activity-based reimbursements. DRG-weights are used as a measure of hospital activity as function of the patients’ diagnosis, comorbidities, hospital bed stay and procedure/treatment, and designed to reflect 50% of the national average cost of treatment. The sum of all DRG weights produced within a hospital region, determines the share of the activity-based reimbursement the hospital receives. Each of the four trusts then distribute their budget across smaller administrative hospital regions We measure healthcare utilisation as the use of healthcare services by the populations living within each of the 19 hospital regions in Norway.

Block grants are divided based on a resource allocation formula that largely considers the number of inhabitants in each region, adjusted for population characteristics such as age, socioeconomic conditions, mortality, and climate [17]. From 2011–2015, once these adjustments were considered, the aggregate DRG-weight production per capita varied by only 4% to 11% [18].

Given such small variations at the aggregate level, the observation of large variations for specific treatment rates suggests a substitution effect must exist at some level of service delivery–i.e. a region with a treatment rate *higher* than the national average for one specific treatment must have a treatment rate *lower* than the national average for at least one other treatment. Hence, when explaining variation for one specific treatment, we consider the association between the utilisation of other treatments. Two treatments performed by surgeons with the same specialisation are necessarily substitutes, from a supply perspective–a surgeon can only treat one patient at the time. However, budgets for a particular medical specialty may differ across regions. If hospitals increase the budgets for a particular medical specialty where they have comparative advantages, a positive correlation between treatments with similar characteristics can occur.

### 2.1 Treatments with similar characteristics

We identify pairs of treatments in which both treatments are performed by surgeons with the same specialty, and the patients experience loss of HRQoL in the same dimension. The four pairs are: (1) meniscus and shoulder surgery (acromion resection); (2) lumbar spinal stenosis (LSS) and lumbar disc herniation (LDH); (3) tonsillectomy and ear drain (tympanostomy tube), and; (4) heavy eyelids and cataracts.

Common to all these treatments is uncertainty regarding when to treat, opening for practitioner bias or patient preferences to influence the decision-making. This increases the likelihood of finding unwarranted variation [9]. All treatments have alternative, non-invasive treatment options and are considered primarily elective–i.e. provided at hospital convenience. However, as with all invasive treatments, some patients have a significant loss of health-related quality of life and is therefore handled as emergency care patients. S1 Table present the ratio of emergency care treatments provided for each surgery. In general, emergency care patients exhibit a more obvious need for care [10] and should therefore be considered qualitatively different from elective care treatments. Hence, it is reasonable to distinguish between those who received emergency care treatment and those who received elective treatment. A summary of key characteristics for each treatment is presented in Table 1.

**Table 1:** Key characteristics of the treatments considered.

	Speciality	Age Mean (IQR)	Quality of life deterioration
**Meniscus**	Orthopedic	49 (40–60)	Joint Pain
**Shoulder**	Orthopedic	54 (47–61)	Joint Pain
**LSS**	Neurologi/Orthopedic	61 (52–73)	Back Pain
**LDH**	Neurologi/Orthopedic	46 (37–55)	Back Pain
**Tonsil**	Ear, Nose, Throat	13 (4–20)	Future infection
**Ear**	Ear, Nose, Throat	12 (3–8)	Future infection
**Eye lids**	Ophthalmologist	61 (53–69)	Vision
**Cataracts**	Ophthalmologist	75 (69–82)	Vision

Note: IQR = Interquartile range, LSS = Lumbar Spinal Stenosis, LDH = Lumbar Disc Herniation.

Treatments in the first pair, meniscus and shoulder surgery, are similar in the sense that they are carried out by doctors with the same surgical specialty (orthopaedic) and in the same ward. The age and sex composition of patients are similar, and the recovery period is similar. Furthermore, the health gain from both treatments is generally considered low [19,20].

Treatments in the second pair, LSS and LDH, are performed by both orthopaedic surgeons and neurosurgeons, and in this sense, they are somewhat overlapping with the first pair. Both conditions involve a significant loss of HRQoL. Clinically significant health gains have been observed among 65% of treated LDH patients [21] and 74% of LSS patients [22].

The third pair of treatments, tonsillectomy and ear drain, is associated with paediatric care and is performed by ear, nose, and throat (ENT) surgeons. It is unclear what positive effect ear drain surgery has [23], while tonsillectomies have been found to increase the risk of respiratory illness in the long term [24].

The fourth pair of procedures is primarily for older patients who have the option to adapt and live with their conditions instead of undergoing surgery. The efficiency of cataract surgery is questionable [25], while heavy eyelid surgery is considered primarily cosmetic.

## 3. Data

We used data from the Norwegian Patient Register (NPR) and Statistics Norway for 2010–2015. The NPR data contains demographic and hospital admission information for all surgeries financed by the government (see Table 2 for descriptive statistics). To identify spine surgery patients, we used the combination of procedural codes (NCPS) and diagnostic codes (ICD-10) developed by the Norwegian Registry for Back Surgery (second pair of treatments). For the other treatments, we used NCPS and ICD-10 combinations as defined by the Centre for Clinical Documentation and Evaluation in their *Day Surgery Atlas*, *2011–2013* (www.helseatlas.no/en). A total of 548,696 surgeries were included in our dataset.

**Table 2:** Variation coefficient for the entire period and highest/lowest variation coefficient across all years during the study period.

		Meniscus	Shoulder	Stenosis	Disc	Tonsil	Ear	Eye	Cataracts
**Var Coef tot**		1.96	3.11	2.16	2.61	2.1	2.6	2.4	1.5
**Var by year**	Min	2.81	2.81	2.06	2.36	1.96	2.79	2.4	1.7
	Max	3.69	3.69	2.73	3	2.71	3.38	3.7	2.3

Note: Variation coefficients were calculated by dividing the mean of the three highest rates by the mean of the three lowest rates.

Statistics Norway’s database contains the age and sex distribution in Norwegian municipalities, which we used to calculate standardised procedure rates for each hospital region.

The data collection was done by the NPR and Statistics Norway, and no patients consent was required according to Norwegian law. The merging and handling of data was approved by the Regional Ethics Committee [Ref: 2016/2059], the Norwegian Data Protection Authority [Ref: 17/00429–2/SBO] and the NPR [Ref: 17/12072–9]

### 3.1 DRG as an outcome measure

Each combination of diagnosis and procedural codes results in a specific DRG. Each DRG is assigned a weight, where one DRG is a reflection of the national average hospital costs of treating a patient with the given diagnosis and procedural code. Aggregate DRG-weight production per capita does not vary significantly across Norwegian hospital regions, which are subject to strict government-imposed distributive funding mechanisms. One should assume that they have the capability of producing the same DRG rates within the set of treatments we consider.

To account for variation in need, we calculated treatment rates with direct standardisation, adjusted for sex and age, using eight strata for age. Based on the treatment rates, we calculated the DRG rates–i.e., the resources spent on each treatment per capita, as measured by DRG weights.

Using DRG rates rather than standardised treatment rates, we compared the resources invested in treatment. We also accounted for the variation in the unit cost associated with each treatment. For example, within LDH surgery, there are multiple techniques commonly used to perform the surgery and variations in the diagnoses. This results in different unit costs for patients treated for LDH. Furthermore, the DRG weights are subject to change year on year. Hospitals have been demonstrated to be sensitive to these changes, which will be accounted for in the DRG-rate.

Hence, we calculate DRG per capita per treatment, region, and year according to the formula:

$${DRG}_{ijt}=\sum_{Code=1}^{X}\left(\frac{n_{ijt}^{Code}}{N_{ijt}^{tot}}*{weight}_{t}^{Code}\right)*{Rate}_{ijt}^{Adj}$$

Where DRG per capita for treatment *i* in region *j* during year *t* is calculated based on the ratio of patients with a combination of diagnostic and procedural codes, resulting in the given DRG *code* multiplied by the standardised treatment rates and the DRG *weight*.

Private specialists do not receive DRG reimbursements but are compensated according to actual cost of treatment. These costs were not available to us. Hence, patients treated by private specialists were assigned the weighted average DRG from public hospitals within their region of residence. Such transformations have previously been conducted in Norwegian Official Reports [17,26].

### 3.2. Analysis

We calculated the variation coefficients by dividing the mean of the three highest rates by the mean of the three lowest rates.

Most variation in expected population need is accounted for through age and sex standardisation [17]. However, other factors such as preferences, economies of scale, or spill-over effects in service provision are unobservable for us. Hence, using DRG rates from standardised treatment rates, we aim to estimate the substitution effects as a latent variable.

To estimate these latent variables, we applied PCA [27]. This method is used to find linear representation of all variables in a dataset, making it suitable for data with collinearity. These variables are expressed as eigenvectors from the covariance matrix of the included variables. To demonstrate this process, say *X* contains the column vectors of the eight treatments in the dataset (*X* = [*x*_1_,*x*_2_,…*x*_8_]). The column vector *B* would be a linear representation of *X* such that *B*′*B* = 1.

The variance in the data can then be expressed as

$$Var[B^{\prime }X]=E{\left[B^{\prime }X\right]}^{2}$$

Substituting *X* with its covariance matrix, *C* gives

$$Var[B^{\prime }X]=B\prime CB$$

Then, to find B, we solve the Lagrangian

$$L=B^{\prime }CB-\lambda (B^{\prime }B-1)$$

The first order condition of *L* with respect to *B* is

$$\frac{\partial L}{\partial B}=2CB-2\lambda B=0$$

which can be simplified to *CB* = *λB* –i.e. *B* is the eigenvector for the covariance matrix of *X*. The eigenvectors are a set of vectors associated with a linear system of equations. Therefore, using the eigenvectors, one can reproduce the data structures of the original data. An accompanying eigenvalue *λ* describe the scaling, or how much weight should be placed on each eigenvector. Since the sum of the eigenvalues is equal to one, the eigenvalue describes the amount of variation described in one eigenvalue.

The eigenvector with the highest eigenvalue is used as the principal component, while the eigenvector with the second highest eigenvalue is used as the second principal component, and so on. Hence, by ranking the eigenvectors by their accompanying eigenvalues, we can express the most important dimensions of the data in lower dimensional space. This allowed us to evaluate the correlation across all DRG rates. PCA is sensitive to scaling of the data; hence, all DRG rates were standardised to a Z-score.

Applying the PCA to the standardised DRG rates provides insight into the treatment profiles–i.e. how the DRG rates relate to each other. Moreover, theory dictates that we should find a higher degree of variation in the elective treatments. Hence, we provide separate analyses on elective and emergency care treatments. We define elective care treatments as those for which patients waited more than 24 hours after referral from a GP.

To determine whether provision of one treatment results in reduced supply of another treatment, i.e., a substitution effect, we considered the relative annual change in DRG rates. By considering the relative change in DRG-rates, the effect of initial differences in nominal budgets are eliminated. We estimated the relative differences by first calculating the natural logarithm of annual DRG production in each region, and then using the differences between the logarithmic values as a measure of relative annual change (first difference log). We did this for all treatments within each region in each year and conducted the same analysis.

While it is possible to quantify the associations between treatments through principal component regression, we have not found suitable implementations that account for both region- and time-specific fixed effects. Suggestions have been made to incorporate necessary fixed effects [28], but these are not suitable for our dataset.

## 4. Results

**Fig 1:**
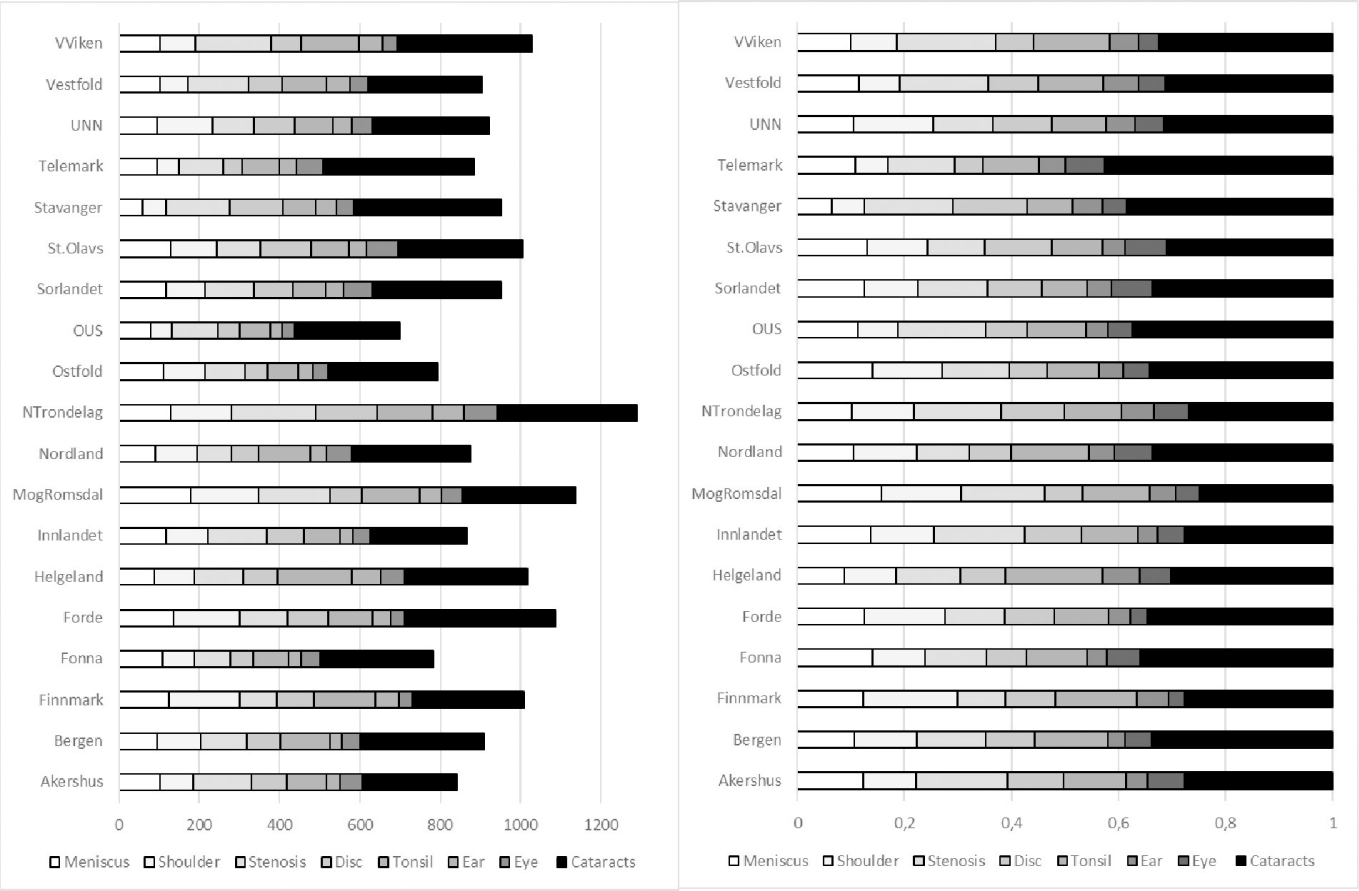
Visualisation of mean DRG weight production per 100 000 capita, per region per condition (2010–2015). Nominal weights to the left, each treatment as percentage of total production to the right.

Fig 1 show the mean DRG rate per 100,000 capita by treatment and region. S1 Table provides the ratio of elective treatments in each region for each treatment. In Table 2, the variation coefficients for the entire period is provided along with the highest and lowest variation coefficient for each year during the study.

The variation in mean DRG rates ranges from 1.5 for *cataracts* to 3.11 for *shoulder*. Note how the mean variation coefficients in DRG rates for *meniscus* and *cataracts* are below the variation coefficients for any given year. We interpret this to reflect less systemic variation in these two treatments compared with the other six treatments. This can be illustrated by a simple example: If we observe two regions A and B over two years and their respective treatment rates are {10, 2} and {2,10}, then the variation coefficient for a single year would be 10/2 = 5, while the mean would be ((10+2)/2) / ((2+10)/2) = 1 –i.e., no persistent variation over time.

**Fig 2:**
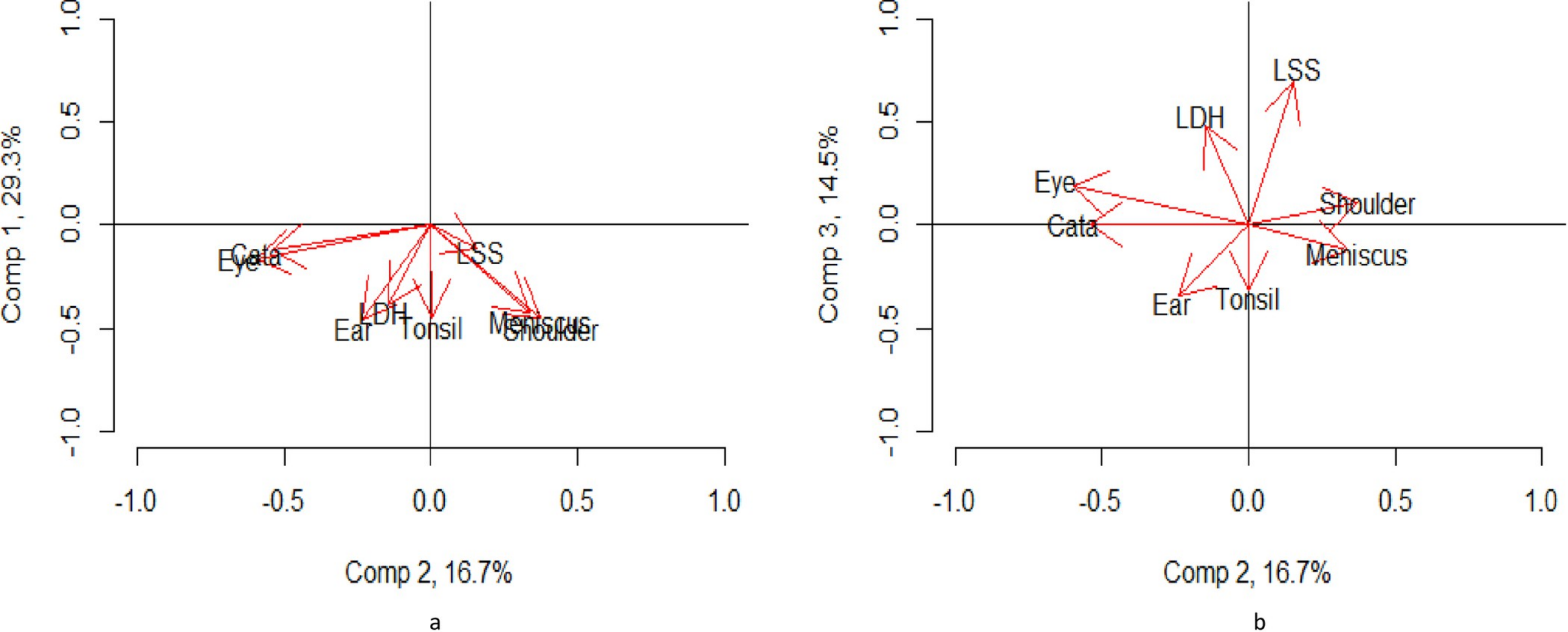
Visual representation of PCA of DRG weight production for eight surgical treatments per 100,000 capita over six years. Left: First and second components; right: Second and third components.

The loading scores from the first PCA is presented in S2 Table, while the most important components is depicted in Fig 2. The loading scores is a representation of how variation in the DRG-rates for one treatment relates to all other treatment include in the study, decompartmentalised into eight dimensions (equally many dimensions as input variables in the study). Each component is representing a part of the total variation in the dataset, and each component have an accompanying eigenvalue, describing how much of the total variation in the dataset is expressed in a given component. In the principal component (first component), all elements are negative. Thus, all elements indicate the same direction, which should be interpreted as all treatments being positively correlated. This underlying data structure is expressed in the primary component, meaning that the dimension which explains the most variation is related to the ‘size’ of the data [29]. Simply put if treatment rates for one of the included treatments is high in a region, then it is likely high for the other treatments as well. This treatment frequency accounts for 29.3% of the overall variation.

Due to the properties of the principal component, we also focus on the second and third components. In Fig 2, we plotted treatments according to their loading scores to visualise how the variations in the treatments relate to each other. The arrows in Fig 2 represent the direction of variation for each treatment relative to the component. The length of the arrow imply the impact or how much variation is explained for each treatment.

The principal component primarily explains variation in *meniscus*, *shoulder*, *LDH*, *tonsil*, and *ear*. For the second component, it appears that DRG rates are clustered {*eye*, *cataracts*}, {*LSS*, *LDH*, *tonsil*, *ear*}, and {*meniscus*, *shoulder*}. However, when ignoring the ‘size’ component, the plots of the second and third components (Fig 2b) demonstrate how the DRG rates cluster as hypothesised. While the second component clearly differentiates between {*eye*, *cataracts*} and {*meniscus*, *shoulder*}, the third component differentiates between {*LSS*, *LDH*} and {*tonsil*, *ear*}. Thus, while the principal component explains 29.3% of the variation in the data, the second and third components explain another 31.2% (second component 16.7%, third component 14.5%).

**Fig 3:**
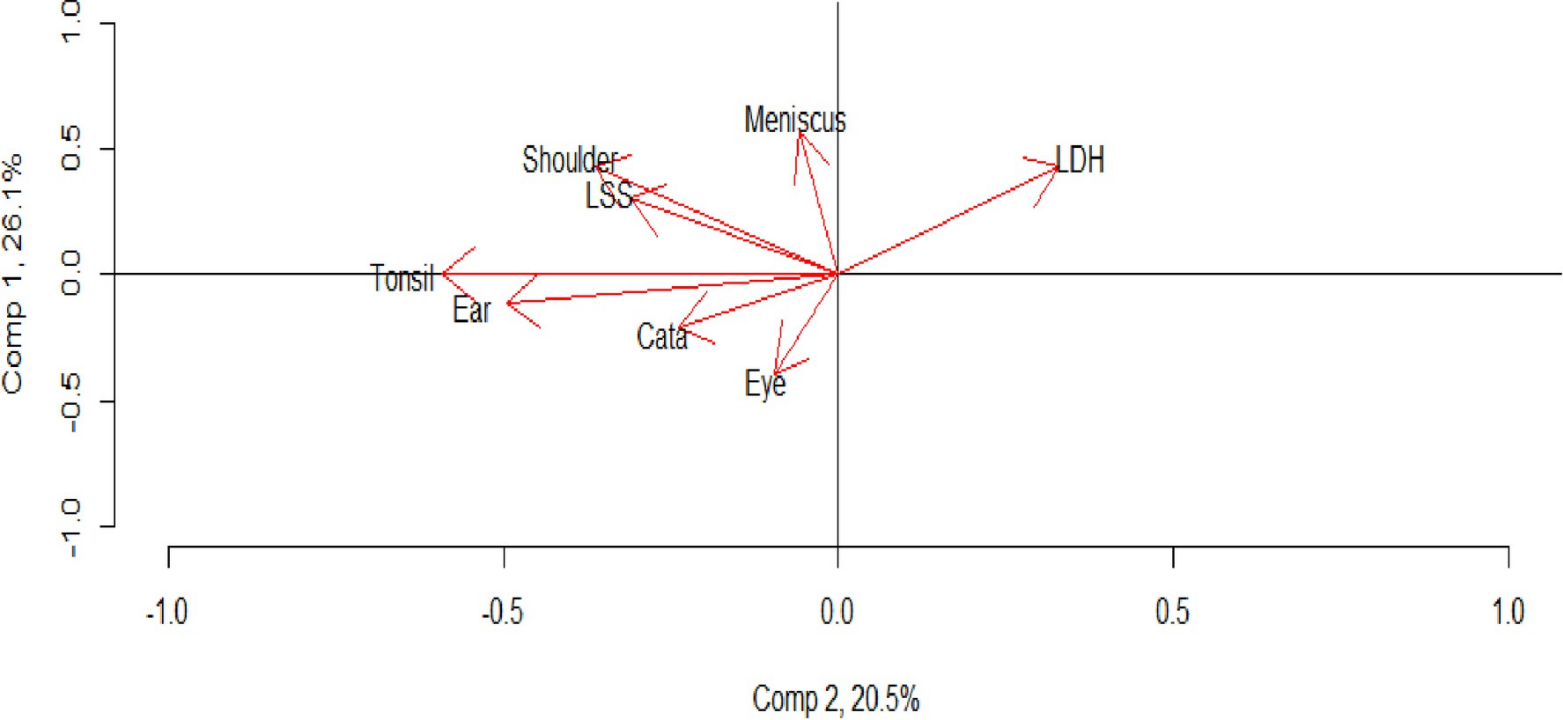
Visual representation of first and second component from PCA of annual change in DRG weight production per capita for eight surgical treatments.

Loading scores from the PCA analysis based on the first difference of the natural logarithm for the rates within each region is presented in S3 Table. As this is the relative change in DRG rates, the size component is now unaffected by possible variations in budget size. The two components with the highest explanatory power are illustrated in Fig 3.

The principal component explains 26.1% of the variation in the data. Moreover, it separates the DRG rates into two groups: {*meniscus*, *shoulder*, *LSS*, *LDH*} and {*ear*, *cataracts*, *eye*}, while none of the variation in tonsillectomy is explained in the principal component. In the second component, we can see in Fig 3 how LDH separates from all other treatments. Note that LDH is the treatment with the highest ratio of emergency care treatments (33.1%).

Loading scores for the relative change in DRG-weight production when only elective care treatments were included is presented in S4 Table. In the principal component, there is a clear separation between {*eye*, *cataracts*} and the other six treatments. The interpretation is that an increase in elective treatments for {*eye*, *cataracts*} is associated with a reduction in elective treatments for the other six treatments. Furthermore, in the second component, {*tonsil*, *ear*} is separated from the six other treatments.

**Fig 4:**
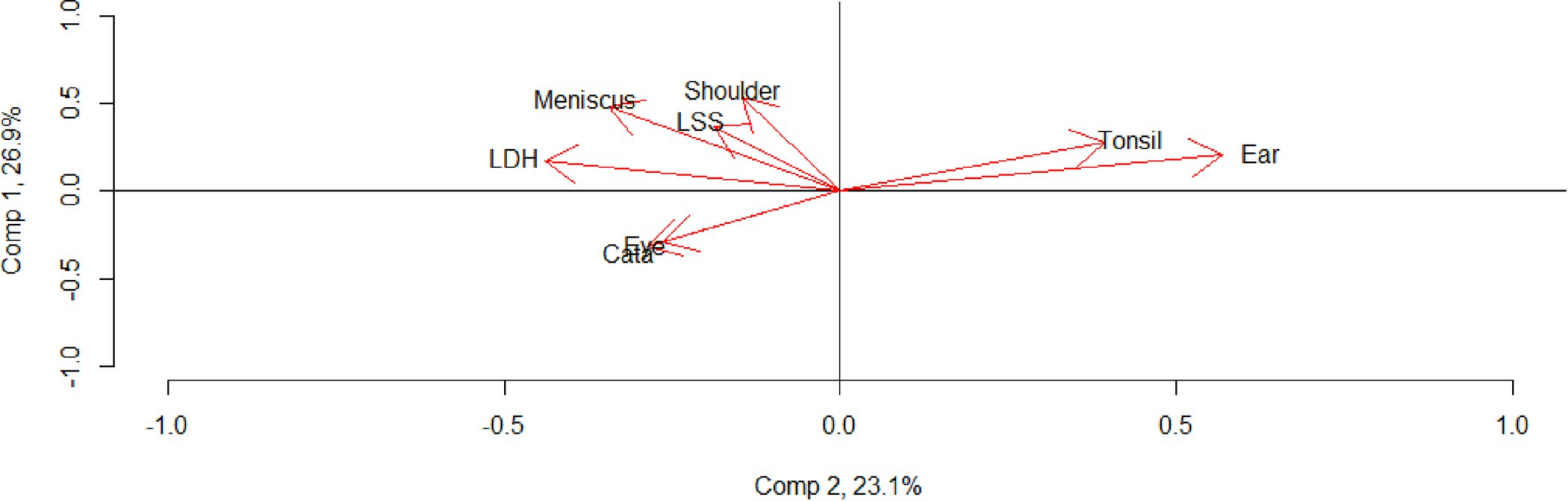
Visual representation of first and second component from PCA of annual change in DRG weight production for elective treatments per capita for eight surgical treatments.

The first two dimensions of the PCA explain, in total, 49% of the variation in elective DRG rates. When plotting the primary and secondary component, as in Fig 4, there is a clear separation between three groups: {*meniscus*, *shoulder*, *LSS*, *LDH*}, {*tonsil*, *ear*}, and {*eye*, *cataracts*}.

The separation is as hypothesized, except for {*meniscus*, *shoulder*} and {*LSS*, *LDH*}. However, orthopaedic surgeons routinely perform LSS and LDH surgeries. Hence, the interpretation of our results is straightforward: for elective care treatments, the substitution in DRG rates occurs *across* medical specialties, and not *within*.

## 5. Discussion

Regional variations are observed for most treatments in specialised care [3]. It has been shown that the variation in a specific treatment is persistent over time [4–6]. In Norway, hospital financing is centrally distributed by a combination of 1) block grants according to regional demographics and historical service provision, and 2) activity-based financing based on DRG-weight production. As a result, there is little variation in aggregate DRG rates across regions [18]. For specific treatments, however, we observe significant regional variations in DRG rates. There is no *ex-ante* reason why we should observe such regional variation, which raises concern of unwarranted regional variations in health service provision.

We have demonstrated a frequency component in the DRG rates. Regions with high DRG rates for a primarily elective care treatment tend to have high DRG rates for other primarily elective care treatments. This component is independent of medical specialty or treatment characteristics. Looking beyond this frequency component, treatments cluster according to medical specialty. Moreover, in comparing the results with and without emergency care treatments (Figs 3 and 4 and S3 and S4 Tables), we have demonstrated the importance of distinguishing between elective and emergency care treatments, and that substitution effects occur *across* medical specialties.

The first contribution of our findings is that 29.3% of the variation in DRG rates could be explained by the treatment frequency in a region, independent of treatment characteristics. Elective care is provided at hospital convenience; therefore, frequency of these treatments should have an inverse correlation with the regional variation in need. The variation in need by region in Norway is reflected by for instance variation in life expectancy [30], and hip fracture repairs [31]. Moreover, patterns of care emerge when removing the frequency component. DRG rates cluster according to medical specialty and the dimension in which loss in HRQoL occurs.

The second contribution pertains to the dynamic model. It is possible that the correlation in treatment pairs is due to regional variations in budgets allocated for surgery (compared to rehabilitation, conservative treatments in specialised care, etc.). Hence, we considered the relative *annual change* in DRG rates in identifying the substitution effect. A pattern of substitution across specialties emerged, with LDH being an outlier. When we included only elective treatments, we found a clear pattern in which the substitution effect occurred across medical specialisations. Our findings are in line with previous studies, that demonstrated how the substitution effect was likely to occur *across* medical specialties [8,32].

The method applied here was developed to detect patterns in data with multi-collinearity, where the outcomes of interest are highly dimensional in nature. However, we have not seen previous studies that have applied it in the context of regional variations. The advantage of PCA is that it reduces dimensionality in data and is therefore suitable should one want to conduct similar analysis with additional treatments. Furthermore, PCA is not subject to omitted variable bias, as parametric system of equations would be. The assumption in analysis is that utilisation of treatment *A* affects utilisation of treatment *B*. Any treatment omitted from our analysis will therefore have the potential to be correlated with both A and B. Without observing all treatments provided, only unrestricted models could be applied. Future studies applying the PCA model can use the same study setup independent of the number of additional treatments available. As only eight treatments from four specialties were included, interpretation from a graphical representation of the results was the most reasonable solution to identify the substitutions. However, should the analysis be extended to n treatments across m specialties, there are multiple clustering techniques which could be applied to identify substitutes. However, as a sensitivity analysis, the analysis was conducted using Seemingly Unrelated Regression [33]. The results provide the same inference as the PCA and are available upon request to corresponding author.

The strengths of the study are that it is based on a national registry including 90–95% of all patients treated with the surgeries under investigation (patients paying out of pocket or with private insurance are not included in NPR). The Norwegian registries have high completeness and are frequently used in scientific research. As for study limitations, there might be economies of scale/efficiency considerations, not accounted for in the study, nor could we account for patient outcomes. Also, we recognize the risk of misclassification in these registries, and we are not able to validate the observations. Furthermore, our analysis would have been improved had we had access to all treatments provided during the study; however, this was unavailable to us. This also apply, had information on the patients’ use of primary and tertiary care been available. Moreover, patients’ use of primary and tertiary care is organized on a municipal level. Therefore, variation in service supply on a municipal level within the hospital regions might have influenced the results. There is also a possibility that classification of emergency care using a 24 hour cut-off has misclassified some patients. DRG-weight assigned to patients treated by private specialists might have affected the results. Lastly, while the DRG rates are age and genders standardized, other omitted regional population characteristics such as level of education, income or GP density could affect who received treatment. Sufficient data on such factor were unavailable to us.

Our results indicate that future studies should distinguish between emergency and elective care treatments. It is well established that there is a higher risk for unwarranted variations in conditions with uncertainty of when and how to treat. Emergency care patients tend to exhibit clearer indications on when to treat [10], and therefore face a different path to treatment. Not taking this into account will in the very least lead to unobserved heterogeneity.

Our results have major implications for policies aimed at reducing unwarranted variations in healthcare systems with activity-based reimbursements. If a policy is directed at a specific treatment, without considering the ratio emergency/elective care and other service provision within the same medical specialty, the policy will likely have unintended implications.

Furthermore, when hospital financing is subject to a regional distribution model based on historical use of service and expected need based on population characteristics. These models tend not to distinguish extensively on types of services used. During the period studied here, the distribution was modelled based on utilisation in 2004–2005 [17], and an updated distribution model was based on utilisation in 2015–2016 [26]. If a region had relatively higher use of healthcare services than other regions when the first model was developed, this higher use was perpetuated from the model. Therefore, developing new distribution models based on use of healthcare services with low uncertainty surrounding how, when and whom to treat are likely a better reflection of the expected need in the population. Some relevant illnesses to be included in such a model might be hip-fracture repair, breast cancer or heart failure.

Furthermore, as treatment frequency is a significant factor for regional variations in utilisation, regional distribution of block grants should be adjusted for the frequency of elective treatments. Policy changes reliant on coding practices could reduce unwarranted variations, such as by separating emergency and elective care. However, this might incentivise strategic coding. Alternative payment systems have been suggested, such as *pay-for-performance*, *episode-of-care payment* and *bundling of payments*. One way to reduce the unwarranted utilisation would be bundling across treatments, in which payment is adjusted according to an interval for DRG-weight production within a specialty relative to total DRG-weight production in a region. For example, DRGs from elective orthopaedic surgery should not be more than X_1_–X_2_ percent of total DRG-weight production for elective surgeries within a region. This will allow the handling of variations in need within specialties without increasing service delivery disproportionally for one specialty.

## 6. Conclusion

There is a significant correlation between population-based regional treatment rates. The substitution between health services occurs *across*, rather than *within*, medical specialties. To make policy interventions to reduce unwarranted variation for specific treatments, the effects in other treatments need to be accounted for.

## Supporting information

S1 Table Ratio of elective treatments by region, by treatment. (DOCX)

S2 Table Loading scores and variance explained by each component from PCA of DRG weights produced per 100,000 capita for eight surgical treatments in each Norwegian hospital region. (DOCX)

S3 Table Loading scores from PCA using the relative change (first diff of log rates) in DRG weight production per 100,000 capita for eight surgical treatments in Norwegian hospital regions. (DOCX)

S4 Table Loading scores from PCA using the relative change (first diff of log rates) in DRG weight production per 100,000 capita of elective treatments for eight surgical treatments in Norwegian hospital regions. (DOCX)
