# Supplementary material for: Related variations: A novel approach for detecting patterns of regional variations in healthcare utilisation rates
Source: PLoS One. 2023 Jun 22;18(6):e0287306. doi: 10.1371/journal.pone.0287306 (PMC10286998; doi:10.1371/journal.pone.0287306)
Supplement: S1 Table — (DOCX) [file pone.0287306.s001.docx]

|  | Meniscus | Shoulder | LSS | LDH | Tonsil | EaR | Eye | Cata |
| --- | --- | --- | --- | --- | --- | --- | --- | --- |
| Akershus | 0.972 | 0.996 | 0.946 | 0.743 | 0.958 | 0.975 | 0.846 | 0.822 |
| Bergen | 0.907 | 0.994 | 0.946 | 0.661 | 0.972 | 0.990 | 0.722 | 0.895 |
| Finnmark | 0.940 | 0.947 | 0.933 | 0.630 | 0.984 | 0.976 | 0.715 | 0.764 |
| Fonna | 0.964 | 0.996 | 0.942 | 0.649 | 0.991 | 0.996 | 0.812 | 0.833 |
| Forde | 0.968 | 0.991 | 0.938 | 0.663 | 0.994 | 0.988 | 0.721 | 0.826 |
| Helgeland | 0.950 | 0.989 | 0.945 | 0.602 | 0.997 | 0.974 | 0.655 | 0.677 |
| Innlandet | 0.983 | 0.997 | 0.935 | 0.756 | 0.993 | 0.989 | 0.834 | 0.868 |
| MogRomsdal | 0.962 | 0.965 | 0.927 | 0.612 | 0.968 | 0.979 | 0.657 | 0.828 |
| Nordland | 0.955 | 0.994 | 0.934 | 0.616 | 0.983 | 0.983 | 0.790 | 0.722 |
| NTrondelag | 0.966 | 0.948 | 0.882 | 0.606 | 0.982 | 0.989 | 0.683 | 0.823 |
| Ostfold | 0.985 | 0.994 | 0.922 | 0.729 | 0.995 | 0.946 | 0.786 | 0.704 |
| OUS | 0.948 | 0.989 | 0.899 | 0.689 | 0.986 | 0.943 | 0.774 | 0.823 |
| Sorlandet | 0.971 | 0.966 | 0.923 | 0.667 | 0.975 | 0.982 | 0.802 | 0.841 |
| St,Olavs | 0.971 | 0.978 | 0.951 | 0.645 | 0.980 | 0.966 | 0.593 | 0.820 |
| Stavanger | 0.925 | 0.993 | 0.918 | 0.646 | 0.985 | 0.994 | 0.808 | 0.864 |
| Telemark | 0.953 | 0.968 | 0.907 | 0.703 | 0.947 | 0.955 | 0.823 | 0.841 |
| UNN | 0.978 | 0.995 | 0.895 | 0.568 | 0.992 | 0.975 | 0.634 | 0.744 |
| Vestfold | 0.985 | 0.996 | 0.954 | 0.748 | 0.997 | 0.982 | 0.809 | 0.689 |
| VViken | 0.985 | 0.998 | 0.957 | 0.777 | 0.993 | 0.991 | 0.711 | 0.832 |

**Table S1:** Ratio of elective treatments by region, by treatment
