# Supplementary material for: Related variations: A novel approach for detecting patterns of regional variations in healthcare utilisation rates
Source: PLoS One. 2023 Jun 22;18(6):e0287306. doi: 10.1371/journal.pone.0287306 (PMC10286998; doi:10.1371/journal.pone.0287306)
Supplement: S2 Table — (DOCX) [file pone.0287306.s002.docx]

**Table S2:** Loading scores and variance explained by each component from PCA of DRG weights produced per 100,000 capita for eight surgical treatments in each Norwegian hospital region.

|  | 1^st^ comp | 2^nd^ comp | 3^rd^ comp | 4^th^ comp | 5^th^ comp | 6^th^ comp | 7^th^ comp | 8^th^ comp |
| --- | --- | --- | --- | --- | --- | --- | --- | --- |
| Meniscus | -0.425 | 0.337 | -0.119 | 0.423 | -0.111 | 0.031 | -0.644 | 0.292 |
| Shoulder | -0.453 | 0.371 | 0.107 | 0.188 | -0.244 | 0.371 | 0.447 | -0.463 |
| LSS | -0.116 | 0.155 | 0.695 | -0.553 | 0.017 | 0.111 | -0.384 | -0.118 |
| LDH | -0.384 | -0.147 | 0.489 | 0.248 | 0.097 | -0.572 | 0.335 | 0.285 |
| Tonsil | -0.448 | 0.001 | -0.313 | -0.542 | 0.041 | 0.221 | 0.248 | 0.543 |
| Ear | -0.458 | -0.237 | -0.343 | -0.200 | 0.306 | -0.359 | -0.212 | -0.555 |
| Eye | -0.162 | -0.595 | 0.192 | 0.286 | 0.389 | 0.586 | -0.078 | 0.031 |
| Cata | -0.125 | -0.541 | 0.004 | -0.061 | -0.820 | -0.026 | -0.118 | -0.036 |
| Proportion of variance | 0.293 | 0.167 | 0.145 | 0.119 | 0.104 | 0.078 | 0.062 | 0.031 |
| Cumulative proportion | 0.293 | 0.460 | 0.606 | 0.724 | 0.829 | 0.907 | 0.969 | 1.000 |

Note: Loading scores from Principal Component Analysis (PCA) of Diagnosis Related Group (DRG) weight production per capita for eight surgical treatments. Each component describes a ratio of variation in the dataset. The proportion of variance explained was determined by the eigenvalues for each component. Meniscus: Meniscus surgery, Shoulder: Shoulder surgery, LSS: Lumbar Spinal Stenosis, LDH: Lumbar Disc Herniation, Tonsil: Tonsillectomy, Ear: Ear drain surgery, Eye: Heavy eye lid surgery, Cata: Cataract surgery, Comp: Component
