# Supplementary material for: Related variations: A novel approach for detecting patterns of regional variations in healthcare utilisation rates
Source: PLoS One. 2023 Jun 22;18(6):e0287306. doi: 10.1371/journal.pone.0287306 (PMC10286998; doi:10.1371/journal.pone.0287306)
Supplement: S3 Table — (DOCX) [file pone.0287306.s003.docx]

Table S3: Loading scores from PCA using the relative change (first diff of log rates) in DRG weight production per 100,000 capita for eight surgical treatments in Norwegian hospital regions

|  | 1^st^ comp | 2^nd^ comp | 3^rd^ comp | 4^th^ comp | 5^th^ comp | 6^th^ comp | 7^th^ comp | 8^th^ comp |
| --- | --- | --- | --- | --- | --- | --- | --- | --- |
| Meniscus | 0.575 | -0.057 | 0.046 | -0.207 | 0.214 | -0.115 | -0.088 | -0.745 |
| Shoulder | 0.428 | -0.364 | 0.18 | -0.408 | 0.153 | -0.34 | 0.195 | 0.555 |
| LSS | 0.303 | -0.31 | 0.433 | 0.113 | -0.571 | 0.494 | -0.202 | 0.037 |
| LDH | 0.427 | 0.33 | -0.129 | 0.248 | 0.493 | 0.42 | -0.308 | 0.341 |
| Tonsil | 0.002 | -0.591 | -0.246 | 0.384 | 0.277 | 0.297 | 0.517 | -0.102 |
| Ear | -0.11 | -0.495 | -0.533 | -0.144 | -0.032 | -0.052 | -0.658 | 0.036 |
| Eye | -0.396 | -0.096 | 0.299 | -0.585 | 0.389 | 0.493 | -0.038 | -0.077 |
| Cata | -0.209 | -0.238 | 0.572 | 0.453 | 0.364 | -0.342 | -0.342 | -0.036 |
| Proportion of variance | 0.261 | 0.205 | 0.163 | 0.109 | 0.098 | 0.063 | 0.053 | 0.048 |
| Cumulative proportion | 0.261 | 0.466 | 0.629 | 0.738 | 0.836 | 0.899 | 0.952 | 1 |

Note: Loading scores from Principal Component Analysis (PCA) of annual change in Diagnosis Related Group (DRG) weight production per capita for eight surgical treatments. Each component describes a ratio of variation in the dataset. The proportion of variance explained was determined by the eigenvalues for each component. Meniscus: Meniscus surgery, Shoulder: Shoulder surgery, LSS: Lumbar Spinal Stenosis, LDH: Lumbar Disc Herniation, Tonsil: Tonsillectomy, Ear: Ear drain surgery, Eye: Heavy eye lid surgery, Cata: Cataract surgery, Comp: Component.
