# Supplementary material for: Related variations: A novel approach for detecting patterns of regional variations in healthcare utilisation rates
Source: PLoS One. 2023 Jun 22;18(6):e0287306. doi: 10.1371/journal.pone.0287306 (PMC10286998; doi:10.1371/journal.pone.0287306)
Supplement: S4 Table — (DOCX) [file pone.0287306.s004.docx]

Table S4: Loading scores from PCA using the relative change (first diff of log rates) in DRG weight production per 100,000 capita of elective treatments for eight surgical treatments in Norwegian hospital regions

|  | 1^st^ comp | 2^nd^ comp | 3^rd^ comp | 4^th^ comp | 5^th^ comp | 6^th^ comp | 7^th^ comp | 8^th^ comp |
| --- | --- | --- | --- | --- | --- | --- | --- | --- |
| Meniscus | 0.486 | -0.345 | 0.22 | -0.129 | 0.012 | -0.196 | 0.155 | -0.719 |
| Shoulder | 0.537 | -0.145 | -0.081 | -0.262 | -0.276 | -0.458 | -0.23 | 0.525 |
| LSS | 0.373 | -0.188 | -0.582 | 0.083 | -0.222 | 0.654 | -0.025 | -0.038 |
| LDH | 0.177 | -0.44 | 0.363 | 0.128 | 0.622 | 0.298 | -0.254 | 0.293 |
| Tonsil | 0.278 | 0.394 | -0.258 | -0.372 | 0.575 | -0.008 | 0.471 | 0.1 |
| Ear | 0.204 | 0.57 | 0.183 | -0.239 | 0.027 | 0.21 | -0.665 | -0.236 |
| Eye | -0.292 | -0.263 | 0.203 | -0.808 | -0.198 | 0.314 | 0.098 | 0.067 |
| Cata | -0.317 | -0.286 | -0.577 | -0.209 | 0.341 | -0.302 | -0.43 | -0.221 |
| Proportion of variance | 0.259 | 0.231 | 0.138 | 0.107 | 0.101 | 0.066 | 0.051 | 0.047 |
| Cummulative proportion | 0.259 | 0.49 | 0.628 | 0.735 | 0.836 | 0.902 | 0.953 | 1 |

Note: Loading scores from Principal Component Analysis (PCA) of change in Diagnosis Related Group (DRG) weight production of elective treatments for eight surgical treatments. Each component describes a ratio of variation in the dataset. The proportion of variance explained was determined by the eigenvalues for each component. Meniscus: Meniscus surgery, Shoulder: Shoulder surgery, LSS: Lumbar Spinal Stenosis, LDH: Lumbar Disc Herniation, Tonsil: Tonsillectomy, Ear: Ear drain surgery, Eye: Heavy eye lid surgery, Cata: Cataract surgery, Comp: Component
